# Simultaneous integrated boost intensity-modulated radiotherapy post breast-conserving surgery: clinical efficacy, adverse effects, and cosmetic outcomes in breast cancer patients

**DOI:** 10.1007/s12282-024-01588-0

**Published:** 2024-05-05

**Authors:** Yong-qiang Bao, Teng-hua Yu, Wei Huang, Qing-feng Mao, Gan-jie Tu, Bin Li, An Yi, Jin-gao Li, Jun Rao, Huai-wen Zhang, Chun-ling Jiang

**Affiliations:** 1 Department of Radiation Oncology, Jiangxi Cancer Hospital, The Second Affiliated Hospital of Nanchang Medical College, Jiangxi Cancer Institute, Nanchang, 330029 Jiangxi China; 2 Medical Oncology, Nanchang People’s Hospital, Nanchang People’s Hospital Affiliated of Nanchang Medical College, Nanchang, 330009 Jiangxi China; 3 Department of Radiation Oncology, Shandong Cancer Hospital and Institute, Shandong First Medical University and Shandong Academy of Medical Sciences, Jinan, 250117 Shandong China; 4 Key Laboratory of Personalized Diagnosis and Treatment of Nasopharyngeal Carcinoma, Medical College of Nanchang University, Nanchang, 330029 Jiangxi China

**Keywords:** Breast cancer, Breast-conserving surgery, Simultaneous integrated boost intensity-modulated radiotherapy, Clinical efficacy, Adverse effects

## Abstract

**Background:**

Simultaneous integrated boost intensity-modulated radiotherapy (SIB-IMRT) is an innovative technique delivering a higher dose to the tumor bed while irradiating the entire breast. This study aims to assess the clinical outcomes, adverse effects, and cosmetic results of SIB-IMRT following breast-conserving surgery in breast cancer patients.

**Methods:**

We conducted a retrospective analysis of 308 patients with stage 0–III breast cancer who underwent breast-conserving surgery and SIB-IMRT from January 2016 to December 2020. The prescribed doses included 1.85 Gy/27 fractions to the whole breast and 2.22 Gy/27 fractions or 2.20 Gy/27 fractions to the tumor bed. Primary endpoints included overall survival (OS), local–regional control (LRC), distant metastasis-free survival (DMFS), acute and late toxicities, and cosmetic outcomes.

**Results:**

The median follow-up time was 36 months. The 3-year OS, LRC, and DMFS rates were 100%, 99.6%, and 99.2%, respectively. Five patients (1.8%) experienced local recurrence or distant metastasis, and one patient succumbed to distant metastasis. The most common acute toxicity was grade 1–2 skin reactions (91.6%). The most common late toxicity was grade 0–1 skin and subcutaneous tissue reactions (96.7%). Five patients (1.8%) developed grade 1–2 upper limb lymphedema, and three patients (1.1%) had grade 1 radiation pneumonitis. Among the 262 patients evaluated for cosmetic outcomes at least 2 years post-radiotherapy, 96.9% achieved excellent or good results, while 3.1% had fair or poor outcomes.

**Conclusions:**

SIB-IMRT after breast-conserving surgery in breast cancer patients demonstrated excellent clinical efficacy, mild acute and late toxicities, and satisfactory cosmetic outcomes in our study. SIB-IMRT appears to be a feasible and effective option for breast cancer patients suitable for breast-conserving surgery.

## Highlights

1. This study evaluated clinical outcomes, side effects and cosmetic results after SIB-IMRT.
2. The three-year OS, LRC, and DMFS were 100%, 99.6%, and 99.2%, respectively.
3. 96.9% of 262 patients achieved good cosmetic results after 2 year radiotherapy.

## Introduction

Breast cancer has become the most prevalent cancer among women, accounting for 11.7% of all global cancer cases [1]. This increase emphasizes the necessity for effective and efficient treatment strategies. The evolution of diagnostic and therapeutic technologies has facilitated the early detection of breast cancer, particularly in its early stages. With the development of diagnosis and treatment technology, more and more early-stage breast cancer patients can be detected in time. Breast-conserving surgery (BCS) followed by whole-breast irradiation (WBI) has become the main treatment method, as it has been proven to provide at least equal effects in local control rate and overall survival time as mastectomy [2, 3]. However, the traditional whole-breast radiotherapy (RT) regimen, which spans approximately 5 weeks with an additional 1–2 weeks for tumor bed boost, presents challenges such as prolonged treatment duration and increased risk of local tumor recurrence [4].

Despite the established benefits of post-BCS radiotherapy, a significant proportion of patients opt out of this treatment due to various reasons including the length of treatment, perceived discomfort, and economic factors [5]. This highlights an urgent need to refine breast radiotherapy approaches to make them more patient-friendly by reducing treatment duration, lowering costs, and minimizing both acute and late toxicities, thereby making this essential treatment more accessible to a broader patient population [6–9].

The advent of Intensity-modulated radiation therapy (IMRT) has revolutionized radiotherapy by optimizing the dose distribution to the target while sparing surrounding normal tissues, thus mitigating some extent of radiotherapy-induced toxicity. In tandem with the development of IMRT, Simultaneous integrated boost (SIB) technology has been introduced. SIB offers several treatment enhancements such as increased efficacy via higher single-dose irradiation to high-risk areas, optimal and uniform dose distribution within the target area, and a reduction in the number of radiotherapy sessions required [10, 11]. However, the effectiveness and safety of SIB as an adjuvant radiotherapy approach have not been comprehensively established due to the lack of extensive randomized controlled trials, leading to some controversies in clinical guidelines [12, 13].

In light of these considerations, there is a pressing need to evaluate the clinical efficacy, early and late radiotherapy-related adverse reactions, and cosmetic outcomes of SIB in the context of breast cancer treatment. This study aims to fill this gap by conducting a comprehensive retrospective analysis of stage 0–III breast cancer patients who underwent breast-conserving surgery at our institution. We endeavor to provide robust data and analysis to inform future guidelines and clinical practices in the treatment of breast cancer.

## Methods

### Patient selection

This retrospective study analyzed 308 breast cancer patients treated from January 2016 to December 2020. The median age was 45.1 years, ranging from 24 to 72 years. We included patients diagnosed with stage 0–III breast cancer who underwent breast-conserving surgery followed by radiation therapy. The radiotherapy protocols were in line with the standards set by the Radiation Therapy Oncology Group (RTOG) and the European Society for Radiotherapy & Oncology (ESTRO). Systemic treatments were administered according to the National Comprehensive Cancer Network (NCCN) guidelines. Patients were excluded if they had positive surgical margins, bilateral breast cancer, any other malignancy within the past 5 years, non-adherence to radiotherapy or chemotherapy protocols, insufficient follow-up data, concurrent organ dysfunction, or incomplete clinical records. The radiotherapy regimen involved delivering 59.4–59.94 Gy in 2.20–2.22 Gy fractions to the tumor bed volume and 49.95 Gy in 1.85 Gy fractions to the planning target volume, spread over 27 fractions. Radiation therapy was administered using the multibeam IMRT technique, ensuring the prescribed dose covered 95% of the Planning Target Volume (PTV). All IMRT plans utilized 6 MV flattening filter-free photon beams from the Precise linear accelerator (Elekta, Stockholm, Sweden), with the maximum dose rate set to 600 MU/min. Constraints for the Organs at Risk (OARs) were defined as follows: the spinal cord: maximum dose (Dmax) < 3,000 cGy; ipsilateral lung: volume receiving 5% of the dose (V5) < 50%, volume receiving 20% of the dose (V20) < 25%, and mean dose (Dmean) < 1,500 cGy; contralateral lung: V5 < 15% and Dmean < 300 cGy; heart: Dmean < 400 cGy (right side) or Dmean < 600 cGy (left side); the contralateral breast: Dmean < 300 cGy. Regional lymph node radiotherapy was administered to 35.4% of patients, and almost all hormone receptor-positive patients (98.7%) underwent endocrine therapy. Among the HER-2 positive patients, a significant majority (84.8%) received targeted anti-HER-2 therapy. These diverse treatment modalities reflect the tailored approach to breast cancer management based on individual patient and tumor characteristics.

### Adverse effects assessment

Throughout the course of radiotherapy, patients were monitored through weekly consultations, physical examinations, and blood tests to identify and manage any adverse reactions. These reactions were classified and assessed according to the criteria established by the Radiation Oncology Collaborative Group (RTOG) and the European Organization for Research on Cancer Therapy (EORTC) [14]. This thorough and systematic approach ensured the comprehensive recording and management of treatment-related adverse effects, facilitating the evaluation of the therapy's tolerability and safety.

### Cosmetic outcome evaluation

Cosmetic outcomes, an important consideration in breast-conserving therapy, were evaluated using the Harris criteria. This evaluation, conducted during the follow-up visits, categorized outcomes as excellent, good, fair, or poor [15]. This assessment was crucial to understanding the aesthetic results of the treatment, which is a significant factor in patient satisfaction and quality of life.

### Statistical analysis

For statistical analyses, Kaplan–Meier survival plots were generated using SPSS 25.0 software. These plots were utilized to calculate local regional control rates, distant metastasis-free survival rates, and overall survival rates. The choice of statistical methods was aimed at providing a robust and comprehensive analysis of the efficacy of the treatment in terms of disease control and patient survival.

## Results

### Basic patient characteristics

In this cohort of 308 breast cancer patients, the distribution of cancer subtypes was as follows: 21.4% Luminal A, 52.9% Luminal B, 7.5% HER-2 over-expressing, and 18.2% triple-negative. A small subset (2.6%) received neoadjuvant chemotherapy, with half achieving complete remission and the other half partial remission. Axillary debulking was performed in 34.1% of patients, while the majority (84.4%) received adjuvant chemotherapy (Table 1).

**Table 1:** Patient and tumor characteristics

Characteristics	*n*	Percentage (%)
Age at diagnosis (years)		
Mean ± SD	45.1 ± 9.3	
Median (years)	45.1	
Range	24 ~ 72	
≤ 45	162	52.6
> 45	146	47.4
Tumour site		
Left	164	53.2
Right	144	46.8
T-category		
ypT0	3	1.0
ypTis	1	0.3
ypT2	4	1.3
pTis	8	2.6
pT1mi	4	1.3
pT1a-c	183	59.4
pT2	105	34.1
N-category		
ypN0	2	0.6
ypN1	1	0.3
ypN2	2	0.6
pN0	212	68.8
pN1mi	5	1.6
pN1a-c	72	23.4
pN2	12	3.9
pN3	2	0.6
Stage		
0	12	3.9
I	137	44.5
II	143	46.4
III	16	5.2
Histology		
Ductal carcinoma in situ	8	2.6
Invasive carcinoma, non-special type	298	96.8
Invasive carcinoma, special type	2	0.6
Grade		
1	26	8.4
2	146	47.4
3	109	35.4
Not reported	27	8.8
Lymphovascular invasion		
No	294	95.5
Yes	14	4.5
Estrogen receptor		
Negative	76	24.7
Positive	232	75.3
Progesteron receptor		
Negative	95	30.8
Positive	213	69.2
HER2 receptor		
Negative	243	78.9
Positive	65	21.1
Axillary lymph node		
SLNB	239	77.6
ALND	68	22.1
Radiotherapy	5	1.6
Radiotherapy to the superior and inferior clavicular regions		
No	109	35.4
Yes	199	64.6
Internal mammary lymph node radiotherapy	8	2.6
Neoadjuvant chemotherapy	8	2.6
Adjuvant chemotherapy	260	84.4
Endocrine therapy	230	74.7

*DCIS* ductal carcinoma in situ, *SLNB* sentinel lymph node biopsy, *ALND* Axillary lymph node dissection

### Clinical outcomes

**Fig. 1:**
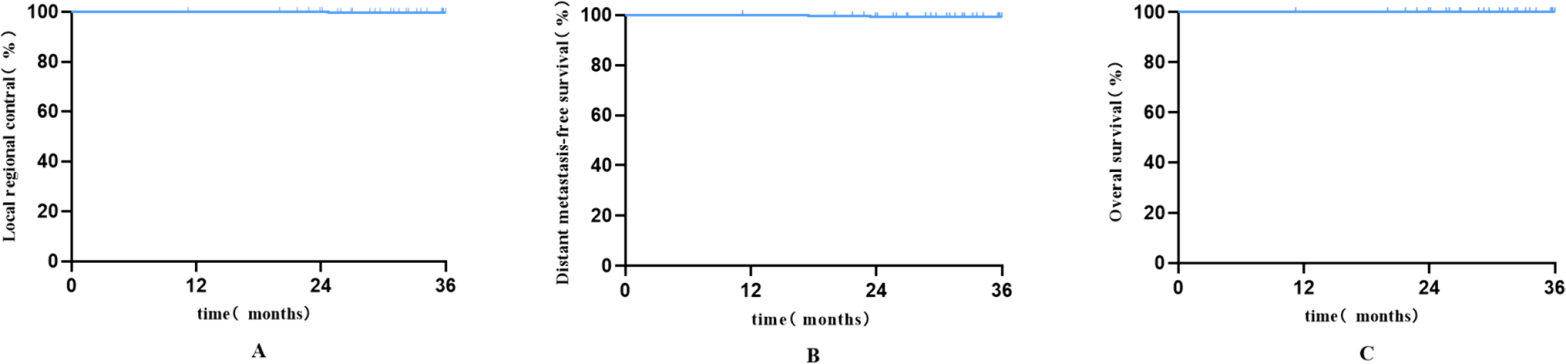
Survival curves (**A** Local area control rate; **B** Distant metastasis free survival; **C** Overall survival rate)

The median follow-up was 42 months, ranging from 11 to 78 months. By November 2022, the follow-up compliance was notably high, with 11.0% lost to follow-up. At 1-year, 3-year, and 5-year milestones, the follow-up rates were 99.7%, 75.6%, and 10.4%, respectively. The study found exceedingly high rates of 3-year local control (99.6%), distant metastasis-free survival (99.2%), and overall survival (100%). These figures underscore the efficacy of the treatment modalities used in this cohort. Survival curves (Fig. 1A–C) illustrate these outcomes.

Local regional recurrence was infrequent, occurring in only 1.09% of patients, and primarily affected axillary and interstitial pectoral muscle lymph nodes, and ipsilateral breast. Distant metastasis was reported in 0.73% of cases, while the mortality rate was also low (0.73%), with one death attributed to distant metastasis from breast cancer and another due to unrelated causes (Table 2).

**Table 2:** Pattern of failures among patients

First event	*n*	Percentage (%)
Local recurrence	1	0.36
Regional recurrence	2	0.73
Local regional recurrence	3(2 triple-negative + 1LuminalA)	1.09
Distant metastasis	2(triple-negative)	0.73
Breast cancer specific death	1	0.36
Death of all causes	2	0.73

### Acute and late radiotherapy reactions

#### Acute radiotherapy adverse reactions

The majority of patients experienced mild acute radiodermatitis (grade 1: 81.2%, grade 2: 10.4%). Grade 1 acute pharyngeal/esophageal reactions were observed in 47.4% of patients. Leucopenia, an important indicator of systemic response, was noted in 25% of patients across grades 1–3 (Table 3).

**Table 3:** Acute radiotherapy adverse reactions

Adverse reactions	*n*	Percentage (%)
Radiodermatitis		
Grade 0	26	8.4
Grade 1	250	81.2
Grade 2	32	10.4
Laryngopharyngitis/esophagitis		
Grade 0	162	52.6
Grade 1	146	47.4
Leukocyte		
Grade 0	231	75.0
Grade 1	33	10.7
Grade 2	36	11.7
Grade 3	8	2.6

#### Late radiotherapy response and cosmetic outcome

Late reactions in the skin and subcutaneous tissue were predominantly mild, with 84.8% experiencing no late reactions (grade 0). Grades 1–3 late reactions occurred in 15.3% of patients. Upper limb lymphedema and radiation pneumonia were rare, observed in 1.8% and 1.1% of patients, respectively. Notably, the cosmetic outcomes post-radiotherapy were predominantly positive, with 96.9% achieving excellent results, affirming the treatment’s effectiveness in preserving aesthetic appearance (Table 4).

**Table 4:** Late radiotherapy adverse reactions and cosmetic

Adverse reactions	*n*	Percentage (%)
Skin and subcutaneous tissue		
Grade 0	229	84.8
Grade 1	32	11.9
Grade 2	8	3.0
Grade 3	1	0.4
Lymphoedema of the upper limbs	5	1.8
radiation pneumonia(grade 1)	3	1.1
Good or excellent cosmetic results (2 years after the completion of radiation therapy)	254	96.9

## Discussion

IMRT-SIB, as a new form of TB supplementary irradiation, has been applied in radiotherapy after BCS. Compared with sequential supplement, IMRT-SIB can improve the dose uniformity within the tumor target area, and reduce the high-dose radiation dose volume received by the affected breast. Especially for deep tumor lesions, imrt-sib technology can further reduce the volume of high-dose areas in the lung and heart. Many studies have shown that the feasibility and dosimetric advantages of IMRT-SIB in the treatment of breast cancer are better than those of the traditional plan [16, 17]. Our study underscores the effectiveness of IMRT-SIB as an adjuvant therapy following breast-conserving surgery, demonstrating remarkable short-term efficacy and excellent cosmetic outcomes [18–20]. The 3-year local control, distant metastasis-free survival, and overall survival rates of 99.6%, 99.2%, and 100%, respectively, not only reinforce the therapeutic value of this approach but also align well with previous literature on IMRT-SIB for breast cancer [21, 22]. McDonald et al. and Bantema-Joppe et al. reported similar findings, thereby validating our study's results [21, 23, 24]. Between March 2011 and August 2015, the NCT 01322854 phase III trial randomized 502 patients to receive either 50.4 Gy in 1.8 Gy single fractions with SIB to the lumpectomy site to a total dose of 64.4 Gy in 28 fractions of 2.3 Gy (IMRT-SIB) or 3D-CRT to the whole breast to a total dose of 50.4 Gy in 28 fractions of 1.8 Gy followed by a seqB to a total dose of 66.4 Gy in 8 fractions of 2 Gy (3D-CRT-seqB) [25, 26]. NCT 01322854 trial (IMRT-SIB) demonstrates that SIB-IMRT delivers a higher dose to the tumor bed while irradiating the entire breast. Our study employs a similar approach, with prescribed doses of 1.85 Gy across 27 fractions to the whole breast and 2.22 Gy or 2.20 Gy across 27 fractions to the tumor bed. Clinical outcomes show that the NCT 01322854 trial achieved a non-inferior 5-year local control rate of 98.7% compared to the control group's 98.3%. Although not explicitly mentioned in our study, excellent clinical efficacy was observed. Both studies reported no significant difference in overall survival (97.1% vs. 98.3%), disease-free survival (95.8% vs. 96.1%), and distant disease-free survival (97.0% vs. 97.8%). Mild acute and late toxicities were reported in both studies. The cosmetic outcomes were not explicitly mentioned in the NCT 01322854 trial, but our study achieved excellent or good results in 96.9% of cases. In conclusion, both studies support the safety and effectiveness of SIB-IMRT for breast cancer patients suitable for breast-conserving surgery. Our study aligns with the NCT 01322854 trial, emphasizing the feasibility and positive outcomes of SIB-IMRT in breast cancer management. A number of other studies also have shown the potential of IMRT-SIB in achieving effective local control, which is a critical factor in breast cancer management [27–29].

The diverse response of different molecular subtypes to IMRT-SIB treatment observed in our study suggests that personalized treatment strategies are essential. Understanding the nuances of how various subtypes, especially more aggressive forms like triple-negative breast cancer, respond to treatment will be crucial in optimizing therapeutic approaches for individual patients. Particularly in understanding and overcoming treatment resistance in this subgroup. This is in line with other studies that have reported higher rates of distant metastases in luminal and triple-negative subtypes [30, 31].

In terms of treatment safety, our study reports a significantly lower incidence of radiation pneumonitis (1.1%) compared to the broader range documented in other studies (4.5–63%) [32, 33]. These patients had only slight imaging changes of pneumonia, but no respiratory symptoms. The radiation dose to the heart is relatively small, so there is no ischemic cardiomyopathy occurred [34]. The reduction of such adverse effects reactions may be mainly attributed to the accuracy of IMRT-SIB radiotherapy dose and the use of advanced improved irradiation technology. This indicates that IMRT-SIB has made great progress in reducing the side effects of radiotherapy [35, 36].

The late cosmetic effect of the whole breast combined with tumor bed concurrent IMRT-SIB has always been the focus of attention. The comprehensive publication of long-term cosmetic results and longer follow-up data for several large randomized controlled phase III trials investigating the application of SIB in adjuvant WBI is still pending [37, 38]. Our findings had a high rate of excellent cosmetic outcomes (96.9% at 2 years) highlight the patient-centric benefits of IMRT-SIB, better than some studies [39, 40]. However, the reliance on subjective assessments by patients and physicians raises questions about the consistency and objectivity of these evaluations. Our study highlights the necessity of incorporating more objective measures in assessing cosmetic outcomes. These detailed analyses of toxicity and cosmetic outcomes could offer new insights into patient experiences and long-term satisfaction with IMRT-SIB, potentially influencing future treatment choices and patient care strategies. Future research should aim to include more standardized and objective measures, such as those employed in the IMRT-MC2 trial, to provide a more comprehensive evaluation of cosmetic outcomes [25, 26, 41]. During the follow-up of this study, it was also found that 5 patients had upper limb lymphedema, which may be caused by comprehensive factors such as local regional radiotherapy and axillary lymph node dissection [42].

In this study, most patients (89.6%) had grade 0–1 acute radiodermatitis, and 10.4% had grade 2. No patients with grade 3 or above skin toxicity were found. This is similar to the findings of Krug et al. [38], but lower than the results reported by Xia et al. [43]. This may be because that most of the patients in this study are hospitalized for radiotherapy, and the skin care during radiotherapy is better, thus reducing the occurrence of higher-level skin toxicity. In addition, relevant studies have shown that the increase of wet desquamation is related to the larger breast volume, and the breast sizes of patients after breast conserving surgery are different in each study, which may be one of the factors that have different incidence of acute skin toxicity in the same type of study [44].

The occurrence of radiation esophagitis is directly related to the radiation dose. Therefore, in the IMRT-SIB for breast cancer, the esophagus must be taken as an OAR for strict dose limit. Our results found that 147 patients (47.4%) had grade 1 acute laryngopharyngitis/esophagitis, which was consistent with the observation of 59.6% grade 1–2 esophageal toxicity after radiotherapy reported by Pasqueer et al. [45], which is slightly lower than that reported by Wang et al. [46]. The reason for this discrepancy might be that their study involved patients who underwent total breast mastectomy and had pathologically positive axillary lymph nodes. The tumor target area in their study is closer to the entrance of the esophagus, resulting in a higher radiation dose to the esophagus. However, our analysis focuses on patients who have undergone breast-conserving surgery, where the target area is relatively farther away, leading to a lower radiation dose to the esophagus.

While our study presents substantial evidence supporting the efficacy and safety of SIB, it is not without limitations. The relatively short follow-up period limits our ability to assess long-term outcomes comprehensively. Although these short-term results are promising, the need for extended follow-up to ascertain long-term efficacy and safety remains paramount [47]. Moreover, the lack of photographic documentation for cosmetic assessments introduces an element of subjectivity that might affect the reliability of these outcomes. The longevity of treatment benefits, potential late adverse effects, and long-term quality of life outcomes are critical components of comprehensive cancer care that require further exploration. To address these limitations, future studies should incorporate longer follow-up durations and objective methods for cosmetic assessment, such as photographic records.

In light of these considerations, our study contributes significantly to the existing body of knowledge, supporting the use of IMRT-SIB in the treatment of breast cancer post-conserving surgery. We advocate for further research to validate these findings in a larger cohort over a more extended period, ensuring that the promising results observed in our study are sustainable and universally applicable. Additionally, patient-reported outcomes and quality of life assessments could be incorporated to provide a more holistic view of the treatment impact. These outcomes are crucial for understanding the patient experience and the broader implications of treatment beyond clinical efficacy.

## Conclusion

In conclusion, our study provides compelling evidence that intensity-modulated radiation therapy with simultaneous integrated boost (IMRT-SIB) following breast-conserving surgery is not only effective in the short term but also yields favorable cosmetic outcomes. The impressive rates of local control, distant metastasis-free survival, and overall survival observed in our cohort are indicative of the potential of IMRT-SIB as a reliable adjuvant therapy in breast cancer management. Furthermore, the high rate of excellent cosmetic outcomes emphasizes the patient-centric advantages of this approach, which are increasingly vital in cancer care.

## Disclaimers

All study participants provided informed consent, and the study design was approved by the appropriate ethics review board. We have read and understood your journal’s policies, and we believe that neither the manuscript nor the study violates any of these. There are no conflicts of interest to declare.

## Data Availability

All data generated and analyzed during this study are included in this published article.

## References

[1] Sung H, Ferlay J, Siegel R (2021). Global Cancer Statistics 2020: GLOBOCAN estimates of incidence and mortality worldwide for 36 cancers in 185 countries. CA Cancer J Clin.

[2] Veronesi U, Cascinelli N, Mariani L (2002). Twenty-year follow-up of a randomized study comparing breast-conserving surgery with radical mastectomy for early breast cancer. N Engl J Med.

[3] Darby S, McGale P, Correa C (2011). Effect of radiotherapy after breast-conserving surgery on 10-year recurrence and 15-year breast cancer death: meta-analysis of individual patient data for 10,801 women in 17 randomised trials. Lancet.

[4] Vujovic O, Cherian A, Yu E, Dar A, Stitt L, Perera F (2006). The effect of timing of radiotherapy after breast-conserving surgery in patients with positive or close resection margins, young age, and node-negative disease, with long term follow-up. Int J Radiat Oncol Biol Phys.

[5] Nattinger A, Hoffmann R, Kneusel R, Schapira MJL (2000). Relation between appropriateness of primary therapy for early-stage breast carcinoma and increased use of breast-conserving surgery. Lancet.

[6] Athas WF, Adams-Cameron M, Hunt WC, Amir-Fazli A, Key CR (2000). Travel distance to radiation therapy and receipt of radiotherapy following breast-conserving surgery. J Natl Cancer Inst.

[7] Voti L, Richardson LC, Reis IM, Fleming LE, Mackinnon J, Coebergh JWW (2006). Treatment of local breast carcinoma in Florida: the role of the distance to radiation therapy facilities. Cancer.

[8] Dreyer MS, Nattinger AB, McGinley EL, Pezzin LE (2018). Socioeconomic status and breast cancer treatment. Breast Cancer Res Treat.

[9] Kumachev A, Trudeau ME, Chan KKW (2016). Associations among socioeconomic status, patterns of care and outcomes in breast cancer patients in a universal health care system: Ontario’s experience. Cancer.

[10] Guerrero M, Li XA, Earl MA, Sarfaraz M, Kiggundu E (2004). Simultaneous integrated boost for breast cancer using IMRT: a radiobiological and treatment planning study. Int J Radiat Oncol Biol Phys.

[11] Singla R, King S, Albuquerque K, Creech S, Dogan N (2006). Simultaneous-integrated boost intensity-modulated radiation therapy (SIB-IMRT) in the treatment of early-stage left-sided breast carcinoma. Med Dosim.

[12] Wenz F, Budach W (2017). Personalized radiotherapy for invasive breast cancer in 2017: National S3 guidelines and DEGRO and AGO recommendations. Strahlenther Onkol.

[13] Smith BD, Bellon JR, Blitzblau R (2018). Radiation therapy for the whole breast: executive summary of an American Society for Radiation Oncology (ASTRO) evidence-based guideline. Pract Radiat Oncol.

[14] Cox JD, Stetz J, Pajak TF (1995). Toxicity criteria of the Radiation Therapy Oncology Group (RTOG) and the European Organization for Research and Treatment of Cancer (EORTC). Int J Radiat Oncol Biol Phys.

[15] Harris JR, Levene MB, Svensson G, Hellman S (1979). Analysis of cosmetic results following primary radiation therapy for stages I and II carcinoma of the breast. Int J Radiat Oncol Biol Phys.

[16] Kestin LL, Sharpe MB, Frazier RC (2000). Intensity modulation to improve dose uniformity with tangential breast radiotherapy: initial clinical experience. Int J Radiat Oncol Biol Phys.

[17] Lin Y, Wang B (2015). Dosimetric absorption of intensity-modulated radiotherapy compared with conventional radiotherapy in breast-conserving surgery. Oncol Lett.

[18] Lee HH, Hou MF, Chuang HY, Huang MY, Tsuei LP, Chen FM, Ou-Yang F, Huang CJ (2015). Intensity modulated radiotherapy with simultaneous integrated boost vs conventional radiotherapy with sequential boost for breast cancer—a preliminary result. Breast.

[19] De Rose F, Fogliata A, Franceschini D, Iftode C, Navarria P, Comito T, Franzese C, Fernandes B, Masci G, Torrisi R, Tinterri C, Testori A, Santoro A, Scorsetti M (2018). Hypofractionation with simultaneous boost in breast cancer patients receiving adjuvant chemotherapy: a prospective evaluation of a case series and review of the literature. Breast.

[20] De Rose F, Fogliata A, Franceschini D (2020). Hypofractionated whole breast irradiation and simultaneous integrated boost in large-breasted patients: long-term toxicity and cosmesis. Clin Breast Cancer.

[21] McDonald MW, Godette KD, Whitaker DJ, Davis LW, Johnstone PAS (2010). Three-year outcomes of breast intensity-modulated radiation therapy with simultaneous integrated boost. Int J Radiat Oncol Biol Phys.

[22] Meng J, Huang W, Mei X (2020). Adjuvant breast inversely planned intensity-modulated radiotherapy with simultaneous integrated boost for early stage breast cancer : results from a phase II trial. Strahlenther Onkol.

[23] Bantema-Joppe EJ, van der Laan HP, de Bock GH (2011). Three-dimensional conformal hypofractionated simultaneous integrated boost in breast conserving therapy: results on local control and survival. Radiother Oncol.

[24] Bantema-Joppe EJ, Vredeveld EJ, de Bock GH (2013). Five year outcomes of hypofractionated simultaneous integrated boost irradiation in breast conserving therapy; patterns of recurrence. Radiother Oncol.

[25] Hörner-Rieber J, Forster T, Hommertgen A (2021). Intensity modulated radiation therapy (IMRT) with simultaneously integrated boost shortens treatment time and is noninferior to conventional radiation therapy followed by sequential boost in adjuvant breast cancer treatment: results of a large randomized phase III trial (IMRT-MC2 Trial). Int J Radiat Oncol Biol Phys.

[26] Forster T, Köhler C, Dorn M (2023). Noninferiority of local control and comparable toxicity of intensity modulated radiation therapy with simultaneous integrated boost in breast cancer: 5-year results of the IMRT-MC2 Phase III trial. Int J Radiat Oncol Biol Phys.

[27] Krug D, Baumann R, Krockenberger K, Vonthein R, Schreiber A, Boicev A, Würschmidt F, Weinstrauch E, Eilf K, Andreas P, Höller U, Dinges S, Piefel K, Zimmer J, Dellas K, Dunst J (2021). Adjuvant hypofractionated radiotherapy with simultaneous integrated boost after breast-conserving surgery: results of a prospective trial. Strahlenther Onkol.

[28] Pfaffendorf C, Vonthein R, Krockenberger-Ziegler K (2022). Hypofractionation with simultaneous integrated boost after breast-conserving surgery: long term results of two phase-II trials. Breast.

[29] Unterkirhere O, Stenger-Weisser A, Kaever A (2023). Single-institution prospective evaluation of moderately hypofractionated whole-breast radiation therapy with simultaneous integrated boost with or without lymphatic drainage irradiation after breast-conserving surgery. Adv Radiat Oncol.

[30] Hwang KT, Kim YA, Kim J (2017). The influences of peritumoral lymphatic invasion and vascular invasion on the survival and recurrence according to the molecular subtypes of breast cancer. Breast Cancer Res Treat.

[31] Metzger-Filho O, Sun Z, Viale G (2013). Patterns of recurrence and outcome according to breast cancer subtypes in lymph node-negative disease: results from international breast cancer study group trials VIII and IX. J Clin Oncol.

[32] Holli K, Pitkänen M, Järvenpää R (2002). Early skin and lung reactions in breast cancer patients after radiotherapy: prospective study. Radiother Oncol.

[33] Kahán Z, Csenki M, Varga Z (2007). The risk of early and late lung sequelae after conformal radiotherapy in breast cancer patients. Int J Radiat Oncol Biol Phys.

[34] Darby SC, Ewertz M, McGale P (2013). Risk of ischemic heart disease in women after radiotherapy for breast cancer. N Engl J Med.

[35] Wei TN, Yeh HL, Lin JF, Hung CC (2023). The clinical outcome of postoperative radiotherapy using hybrid planning technique in left breast cancer after breast-conserving surgery. Cancer Med.

[36] Franceschini D, Fogliata A, Spoto R, Dominici L, Lo Faro L, Franzese C, Comito T, Lobefalo F, Reggiori G, Cozzi L, Sagona A, Gentile D, Scorsetti M (2021). Long term results of a phase II trial of hypofractionated adjuvant radiotherapy for early-stage breast cancer with volumetric modulated arc therapy and simultaneous integrated boost. Radiother Oncol.

[37] Dunst J (2020). Patient reported experience with treatment modalities and safety of adjuvant breast radiotherapy—first results of the randomized HYPOSIB-study. Int J Radiat Oncol Biol Phys.

[38] Krug D, Vonthein R, Schreiber A (2021). Impact of guideline changes on adoption of hypofractionation and breast cancer patient characteristics in the randomized controlled HYPOSIB trial. Strahlenther Onkol.

[39] Choi KH, Ahn SJ, Jeong JU (2021). Postoperative radiotherapy with intensity-modulated radiation therapy versus 3-dimensional conformal radiotherapy in early breast cancer: a randomized clinical trial of KROG 15-03. Radiother Oncol.

[40] Bantema-Joppe EJ, Schilstra C, de Bock GH (2012). Simultaneous integrated boost irradiation after breast-conserving surgery: physician-rated toxicity and cosmetic outcome at 30 months’ follow-up. Int J Radiat Oncol Biol Phys.

[41] Pavy JJ, Denekamp J, Letschert J (1995). EORTC Late Effects Working Group. Late effects toxicity scoring: the SOMA scale. Int J Radiat Oncol Biol Phys.

[42] Morganti AG, Cilla S, Valentini V (2009). Phase I–II studies on accelerated IMRT in breast carcinoma: technical comparison and acute toxicity in 332 patients. Radiother Oncol.

[43] Xia CS, Li MM, Fan M (2017). Clinical outcome of early stage breast cancer treated with simultaneous integrated boost intensity-modulated radiation therapy after breast conserving surgery. Chin J Radiol Med Protect.

[44] Pignol JP, Olivotto I, Rakovitch E (2008). A multicenter randomized trial of breast intensity-modulated radiation therapy to reduce acute radiation dermatitis. J Clin Oncol.

[45] Pasquier D, Le Tinier F, Bennadji R (2019). Intensity-modulated radiation therapy with simultaneous integrated boost for locally advanced breast cancer: a prospective study on toxicity and quality of life. Sci Rep.

[46] Wang DQ, Zhang N, Dong LH (2023). Dose–volume predictors for radiation esophagitis in patients with breast cancer undergoing hypofractionated regional nodal radiation therapy. Int J Radiat Oncol Biol Phys.

[47] Lupattelli M, Palazzari E, Polesel J (2023). Preoperative intensified chemoradiation with intensity-modulated radiotherapy and simultaneous integrated boost combined with capecitabine in locally advanced rectal cancer: long-term outcomes of a real-life multicenter study. Cancers (Basel).

